# Diverse enzymatic chemistry for propionate side chain cleavages in tetrapyrrole biosynthesis

**DOI:** 10.1093/jimb/kuad016

**Published:** 2023-07-08

**Authors:** Richiro Ushimaru, Jiaqi Lyu, Ikuro Abe

**Affiliations:** Graduate School of Pharmaceutical Sciences, The University of Tokyo, Bunkyo-ku, Tokyo 113-0033, Japan; Collaborative Research Institute for Innovative Microbiology, The University of Tokyo, Yayoi 1-1-1, Bunkyo-ku, Tokyo 113-8657, Japan; Graduate School of Pharmaceutical Sciences, The University of Tokyo, Bunkyo-ku, Tokyo 113-0033, Japan; Graduate School of Pharmaceutical Sciences, The University of Tokyo, Bunkyo-ku, Tokyo 113-0033, Japan; Collaborative Research Institute for Innovative Microbiology, The University of Tokyo, Yayoi 1-1-1, Bunkyo-ku, Tokyo 113-8657, Japan

**Keywords:** Tetrapyrrole biosynthesis, Enzyme mechanisms, C–C bond cleavage reactions

## Abstract

Tetrapyrroles represent a unique class of natural products that possess diverse chemical architectures and exhibit a broad range of biological functions. Accordingly, they attract keen attention from the natural product community. Many metal-chelating tetrapyrroles serve as enzyme cofactors essential for life, while certain organisms produce metal-free porphyrin metabolites with biological activities potentially beneficial for the producing organisms and for human use. The unique properties of tetrapyrrole natural products derive from their extensively modified and highly conjugated macrocyclic core structures. Most of these various tetrapyrrole natural products biosynthetically originate from a branching point precursor, uroporphyrinogen III, which contains propionate and acetate side chains on its macrocycle. Over the past few decades, many modification enzymes with unique catalytic activities, and the diverse enzymatic chemistries employed to cleave the propionate side chains from the macrocycles, have been identified. In this review, we highlight the tetrapyrrole biosynthetic enzymes required for the propionate side chain removal processes and discuss their various chemical mechanisms.

**One-Sentence Summary:**

This mini-review describes various enzymes involved in the propionate side chain cleavages during the biosynthesis of tetrapyrrole cofactors and secondary metabolites.

## Graphical Abstract

**Graphical Abstract:**
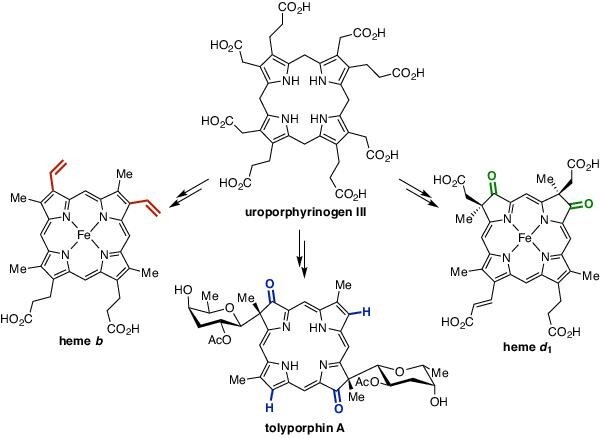

## Introduction

**Fig. 1.**
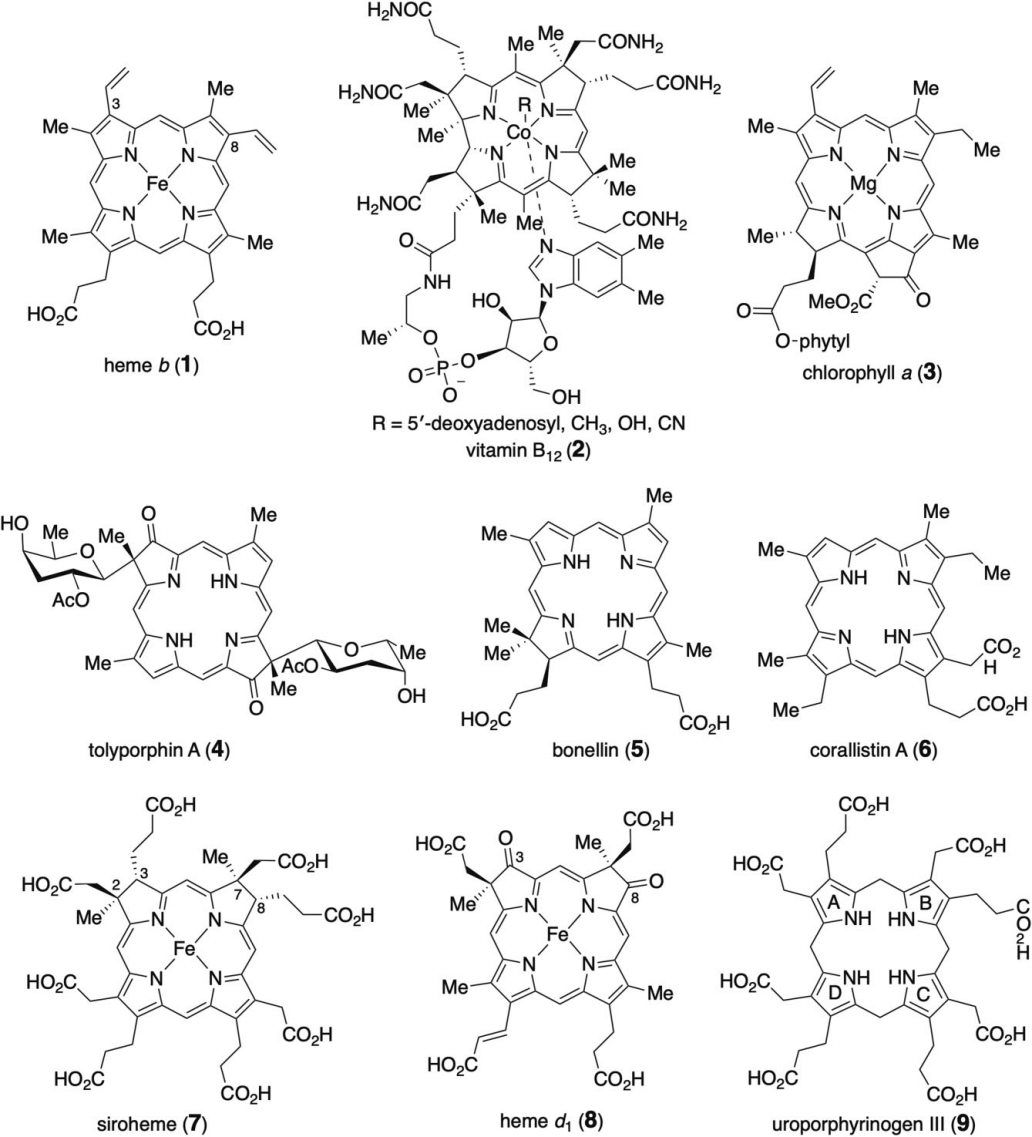
Occurrence of tetrapyrrole cofactors and secondary metabolites.

Tetrapyrroles constitute a large class of natural products that play vital functions in many biological processes due to their unique physicochemical properties (Battersby, 2000). Many natural tetrapyrroles serve as enzyme cofactors. For example, heme *b* (**1**), the most abundant iron-chelating tetrapyrrole, is an enzyme cofactor of peroxidases and the cytochrome P450 family of enzymes for various metabolic reactions (Poulos, 2014) (Fig. 1). It is also an essential component of hemoglobin and myoglobin for oxygen transport and storage in cells, respectively (Fanelli et al., 1964). Vitamin B12 (**2**) is one of the most structurally complex cofactors used for cobalamin-dependent enzymes, which catalyze many radical-mediated reactions in metabolism (Brown, 2005; Gruber et al., 2011). Chlorophyll *a* (**3**) is a magnesium-containing tetrapyrrole derivative that plays a critical role for photosynthesis in plants, algae, and cyanobacteria (Björn et al., 2009; Welschmeyer, 1994). Although many of the known natural tetrapyrroles are commonly used as prosthetic groups for functional proteins involved in essential biological processes, several tetrapyrroles appear to only be produced as metabolites in specific organisms. Such examples include tolyporphin A (**4**) produced by the *Nostocales* cyanobacterium HT-58-2 (Prinsep et al., 1992). Bonellin (**5**) and corallistin A (**6**) are other examples of rare tetrapyrroles isolated from green spoonworm and demosponge, respectively (D'Ambrosio et al., 1989; Pelter et al., 1976). Although the physiological functions of these rare tetrapyrroles (if any) remain unclear, they exhibit cytotoxic activities against human epithelial carcinoma cells and photodynamic activities.

These unique biological properties are attributed to the exceptional diversity and complexity of the molecular architectures of tetrapyrrole natural products. By virtue of their conjugated tetradentate macrocycles, they can chelate various metals at their centers and absorb visible light at certain wavelengths. Although they share the tetrapyrrole core as a common framework, their chemical and physical properties vary depending on the redox level, the peripheral modification of the macrocycles, and the inserted metal ions (Diers et al., 2021). For example, siroheme (**7**) has saturated C2–C3 and C7–C8 bonds and propionate substitutions at C3 and C8 (isobacteriochlorin) instead of the vinyl groups of heme *b* (**1**) at C3 and C8, while heme *d*_1_ (**8**) has two oxo groups at C3 and C8 (dioxo-isobacteriochlorin). These variations of the tetrapyrrole macrocycles modulate the affinity to proteins and regulate the properties of the embedded metal centers (Warren & Scott, 1990). Consequently, siroheme (**7**) serves as a cofactor responsible for the six-electron reduction in sulfite reductases and nitrite reductases (Murphy et al., 1974). In contrast, heme *d*_1_ (**8**) is specifically used by the dissimilatory nitrite reductase cytochrome *cd*_1_ (Fülöp et al., 1995).

Modified tetrapyrroles are biosynthetically derived from a common intermediate, uroporphyrinogen III (**9**), which originates from primary metabolites via several steps. The structure of uroporphyrinogen III (**9**) consists of four pyrrole rings, A–D, each substituted with a propionate side chain and an acetate side chain at the β positions. The macrocycle intermediate undergoes a wide range of chemical modifications, such as methylation, redox reactions, and metalation, in specialized pathways for each tetrapyrrole natural product. Among these modification processes, the propionate side chain cleavage reportedly involves several unique chemistries to introduce C2 (vinyl or ethyl) or C0 (H or oxo) substituents at the pyrrole β sites. In this review, we specifically feature the diverse enzyme chemistries employed to remove the propionate side chains of the macrocycle to generate various modified tetrapyrrole cofactors and natural products. Other tetrapyrrole pathways have been summarized in several excellent reviews (Bryant et al., 2020; Dailey et al., 2017; Heinemann et al., 2008; Layer et al., 2010; Leeper, 1989; Li & Bridwell-Rabb, 2018).

## HemF, HemN, and HemQ in the Biosynthesis of Heme *b*

**Fig. 2.**
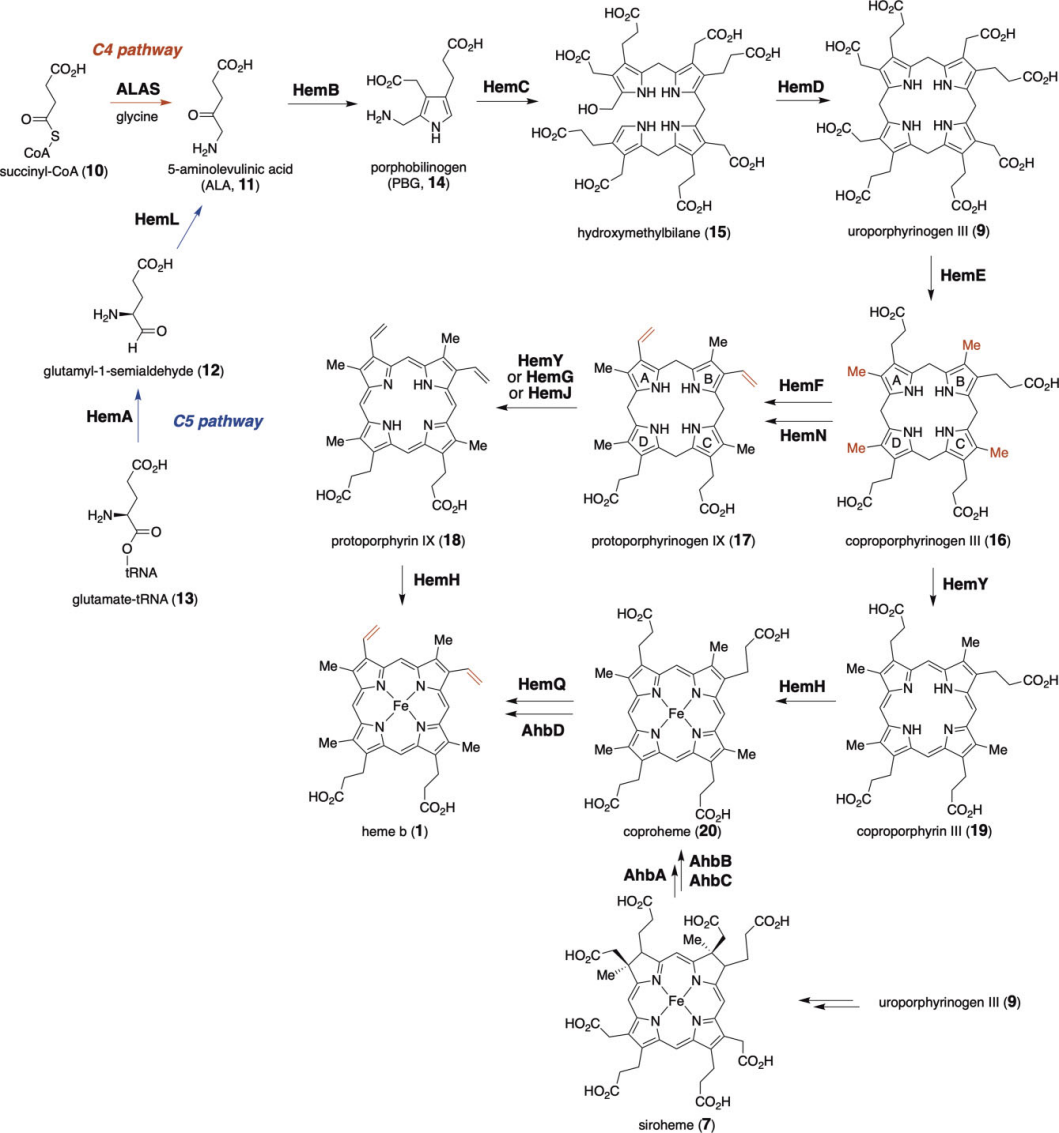
Biosynthesis of heme *b*_1_ (**1**).

The biosynthesis of tetrapyrroles begins with the generation of 5-aminolevulinic acid (ALA, **11**) (Fig. 2). ALA (**11**) is naturally synthesized by two distinct biosynthetic pathways. In mammals, fungi, and alphaproteobacteria, ALA (**11**) is formed via the “C4” pathway by the condensation of glycine and succinyl-CoA (**10**) catalyzed by 5-aminolevulinic acid synthase (ALAS). Plants, archaea, and bacteria utilize glutamyl-tRNA (**13**) to generate ALA (**11**) via the “C5” pathway with two enzymes, glutamyl-tRNA reductase (GtrR, HemA) and glutamate-1-semialdehyde-2,1-aminomutase (GsaM, HemL) (Warren et al., 2009). Subsequently, the cyclic tetrapyrrole intermediate, uroporphyrinogen III (**9**), is assembled via ALA dimerization, pyrrole tetramerization, and macrocyclization, catalyzed by porphobilinogen synthase (HemB) (Jaffe, 1995), porphobilinogen deaminase (PBGD, HemC) (Louie et al., 1992), and uroporphyrinogen III synthase (UROS, HemD), respectively (Mathews et al., 2001; Shoolingin-Jordan, 1995). The acetate and propionate chains on each pyrrole ring in the common intermediate uroporphyrinogen III (**9**) are modified and truncated in downstream pathways to produce various tetrapyrrole cofactors and natural products. For example, in heme *b* biosynthesis, uroporphyrinogen III decarboxylase (UROD, HemE) converts all four acetate side chains into methyl groups to generate coproporphyrinogen III (**16**) via decarboxylation, whereas coproporphyrinogen III oxidase (CPO) facilitates the conversion of coproporphyrinogen III (**16**) to protoporphyrinogen IX (**17**) (Elder & Roberts, 1995). The latter is catalyzed by either the oxygen-dependent HemF in eukaryotes and some bacteria or the oxygen-independent HemN in most bacteria (Troup et al., 1995; Yoshinaga & Sano, 1980a, 1980b). Macrocycle oxidation and iron chelation of protoporphyrinogen IX (**17**) then produce heme *b* (**1**) (Ajioka et al., 2006; Al-Karadaghi et al., 1997; Dailey & Dailey, 1996; Kato et al., 2010; Kobayashi et al., 2014; Lecerof et al., 2000; Möbius et al., 2010). Alternatively, in the monoderm (Gram-positive) bacteria such as Firmicutes and Actinobacteria, the macrocycle oxidation and iron chelation of coproporphyrinogen III (**16**) occur before the propionate side chain is cleaved by coproheme decarboxylase (ChdC, HemQ) (Dailey et al., 2015; Lobo et al., 2015). The conversion of coproheme (**20**) to heme *b* (**1**) can also be catalyzed by the oxygen-independent coproheme decarboxylase AhbD, which is functionally analogous to HemN. The corresponding gene *ahbD* is colocalized with the gene sets of either *hemY*/*hemH*/*hemQ* or *ahbA*/*ahbB*/*ahbC*, which are responsible for the coproheme (**20**) production from siroheme (**7**) (Bali et al., 2011; Dailey et al., 2015). Thus, nature utilizes at least three different classes of enzymes to process the side chain propionate groups and generate the vinyl-substituted tetrapyrroles, as further discussed below (Fig. 2).

**Fig. 3.**
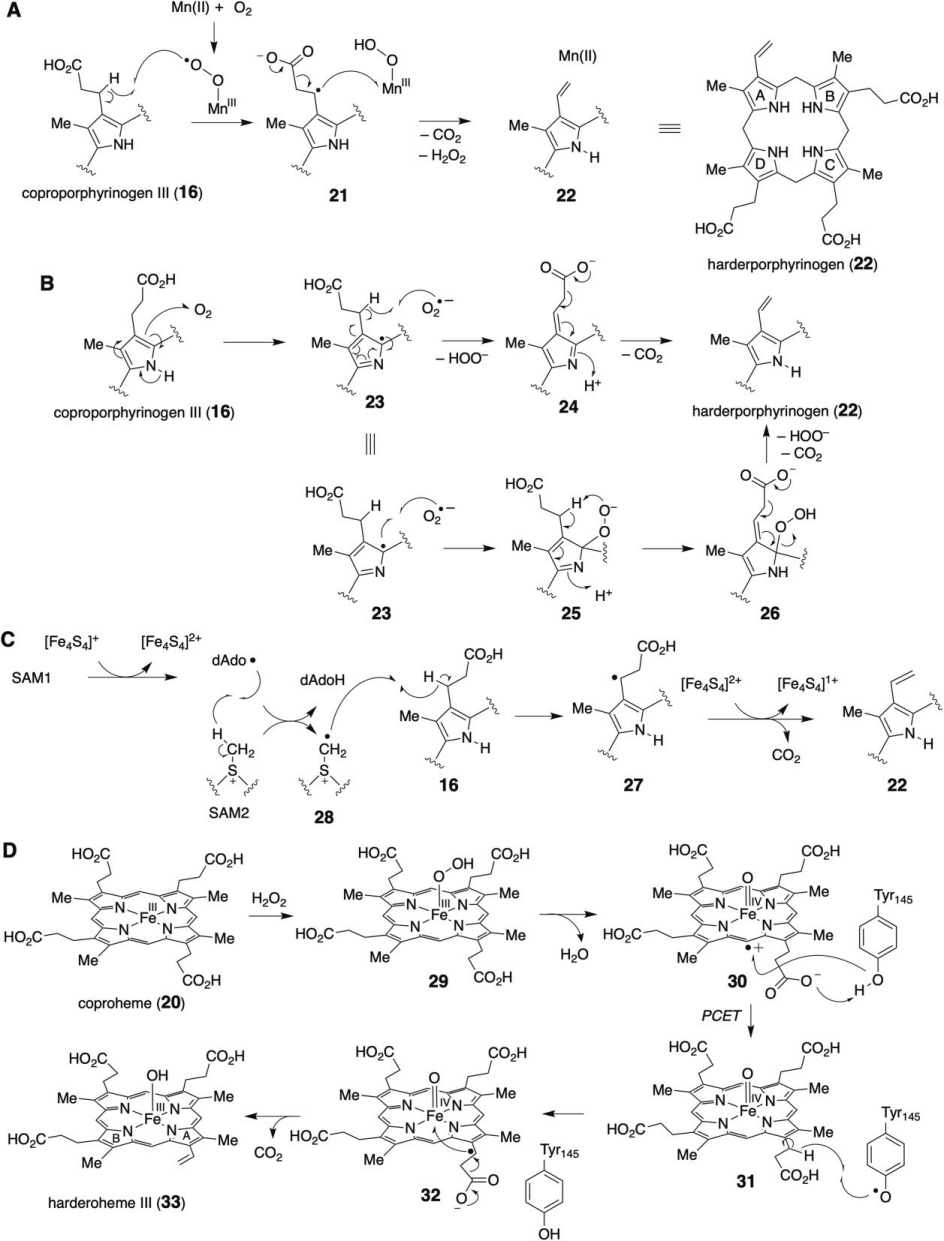
(A) Proposed Mn-dependent mechanism of *E. coli* HemF. (B) Proposed metal-independent mechanism of human HemF. (C) Proposed mechanism of HemN. (D) Proposed mechanism of HemQ.

HemF employs molecular oxygen as an electron acceptor for the oxidative decarboxylation of the propionate side chains on the A and B rings of coproporphyrinogen III (**16**) to generate protoporphyrinogen IX (**17**) (Breckau et al., 2003). Biochemical studies have demonstrated that HemF enzymes from diverse sources exhibit different metal dependencies (Lee et al., 2005). For example, rodent HemF contains copper, yeast HemF contains iron, and human HemF contains no metal for their catalytic reactions, but the roles of the metals have not been fully explored (Kohno et al., 1996; Matringe et al., 1989; Medlock & Dailey, 1996). In a separate study, the participation of manganese in *Escherichia coli* HemF catalysis was also proposed (Breckau et al., 2003). A postulated mechanism for the HemF reaction involves the formation of a Mn(III)-superoxo species upon O_2_ binding that abstracts a hydrogen atom from the β-carbon atom of the propionate group in the substrate, generating the substrate radical **21** (Fig. 3A). The radical intermediate may then undergo decarboxylation and single electron transfer to the Mn(III) species to generate the vinyl group in **22** and the H_2_O_2_ by-product.

Two mechanistic models have been proposed for metal-independent CPOs, such as human HemF. In the Arigoni model (Lee et al., 2005), a single electron transfer from the substrate pyrrole to O_2_ generates the substrate radical **23** and a superoxide ion (Fig. 3B). The superoxide then abstracts a hydrogen atom from the β-carbon of the propionate side chain of the substrate radical **23** to introduce the conjugated imine **24**, which is followed by CO_2_ elimination to form the vinyl group in **22**. In contrast, the Lash model predicts that the pyrrole peroxide anion **25** is first formed, and the β-carbon of the propionate group is then deprotonated in an intramolecular fashion (Lash, 2005) (Fig. 3B). Although the exact catalytic mechanism remains unclear, the resulting intermediate **26** may undergo the elimination of CO_2_ and H_2_O_2_, yielding the vinyl pyrrole intermediate. Nevertheless, the protonation state of the substrate pyrrole in the HemF active site might be critical to initiate the metal-independent decarboxylation reaction with O_2_ (Phillips et al., 2004; Silva & Ramos, 2011).

HemN, an oxygen-independent CPO, is a member of the radical *S*-adenosyl-l-methionine (SAM) protein superfamily (Layer et al., 2005, 2006; Rand et al., 2010). HemN harbors a catalytically important [Fe_4_S_4_] cluster in the radical SAM domain, which mediates the reductive cleavage of SAM to produce methionine and a 5′-deoxyadenosyl radical (dAdo•) (Layer et al., 2003). Previous biochemical studies using deuterium-labeled coproporphyrinogen III (**16**) implied that a hydrogen atom is abstracted from the β-carbon of the propionate group of coproporphyrinogen III (**16**) to form the substrate radical **27**, which then decays to give harderporphyrinogen (**22**) (Layer et al., 2006). Interestingly, the crystal structure of HemN revealed that the protein binds two molecules of SAM (Layer et al., 2003, 2005). *In vitro* assays with deuterium-labeled SAM strongly suggested that dAdo•, originating from the first SAM molecule (SAM1), abstracts a hydrogen atom from the methyl group of the second SAM molecule (SAM2) (Fig. 3C). The existence of the methylene radical **28** during the catalysis was supported by the observation of a conjugate of SAM and the mono-decarboxylated product. Subsequently, the methylene radical species **28** abstracts a hydrogen atom from the β-carbon of the propionate group of coproporphyrinogen III (**16**), which may be followed by decarboxylation involving a single electron transfer to the [Fe_4_S_4_]^2+^ cluster (Ji et al., 2019) (Fig. 3C). It is noteworthy that the binding of two SAM molecules in the active site and the formation of the SAM methylene radical **28** are unique features of HemN, in striking contrast to many other radical SAM-dependent reactions where 5′-dAdo• abstracts a H atom from the substrate (Cheng et al., 2022). AhbD is also a member of the radical SAM enzyme superfamily that transforms coproheme (**20**) to heme *b* (**1**), and its reaction mechanism is believed to be similar to that of HemN (Dailey et al., 2015).

In contrast to HemF and HemN, which accept coproporphyrinogen III (**16**) as a substrate, HemQ (or ChdC) acts on the iron-chelating porphyrin substrate coproheme (**20**) to generate heme *b* (**1**), using H_2_O_2_ as a cosubstrate (Fig. 3D) (Hofbauer et al., 2016a, 2016b; Pfanzagl et al., 2018; Streit et al., 2017). The coproheme (**20**) in the HemQ reaction likely acts as both a substrate and a redox cofactor because HemQ was not active toward Ni-coproheme (Dailey et al., 2015). The reaction of coproheme (**20**) and H_2_O_2_ could generate the Fe(III)-OOH (Compound 0) intermediate **29**. The O–O bond may be cleaved in a homolytic manner to form an Fe(IV)-oxo species and a hydroxyl radical (OH•) (Celis et al., 2015). Alternatively, the heterolytic O–O bond cleavage of **29** may produce the Fe(IV)-oxo porphyrin cation radical species **30** and H_2_O (Celis et al., 2015). The crystal structure of HemQ from the *Listeria monocytogenes* complex with coproheme (**20**) revealed that the protein lacks a distal His–Arg pair and an anionic proximal histidine, which are known to promote the heterolytic cleavage of H_2_O_2_ in other heme-dependent enzymes such as peroxidases. These observations initially implied that the Fe(III)-OOH intermediate **29** is cleaved to generate a hydroxyl radical, which then abstracts a hydrogen atom from the β-carbon of the propionate side chain to initiate the decarboxylation reaction. However, electron paramagnetic resonance (EPR) experiments suggested that a Tyr radical (Tyr145) is formed during the reaction of coproheme (**20**) with H_2_O_2_ (Streit et al., 2018). According to the crystal structure and quantum mechanics/molecular mechanics calculation studies, the Tyr radical is formed via a proton-coupled electron-transfer mechanism involving the propionate side chain of **30** as a proton acceptor (Celis et al., 2017; Tian et al., 2021). A kinetic analysis using a deuterated substrate also indicated that the Tyr radical abstracts the H from the β-carbon of the propionate side chain of **31**, which is partially rate-limiting (Streit et al., 2018). Electron transfer to the Fe(IV) center in **32** and decarboxylation then result in the formation of the first vinyl-substituted intermediate, harderoheme III (**33**). The intermediate **33** may be released from and bound again to HemQ in a reoriented conformation for the next round of propionate decarboxylation on ring B. Alternatively, **33** may rotate by 90° in the active site to produce heme *b*_1_ (**1**) (Liu et al., 2022; Michlits et al., 2020; Milazzo et al., 2019, 2018; Sebastiani et al., 2021).

## NirJ in the Biosynthesis of Heme *d*_1_

**Fig. 4.**
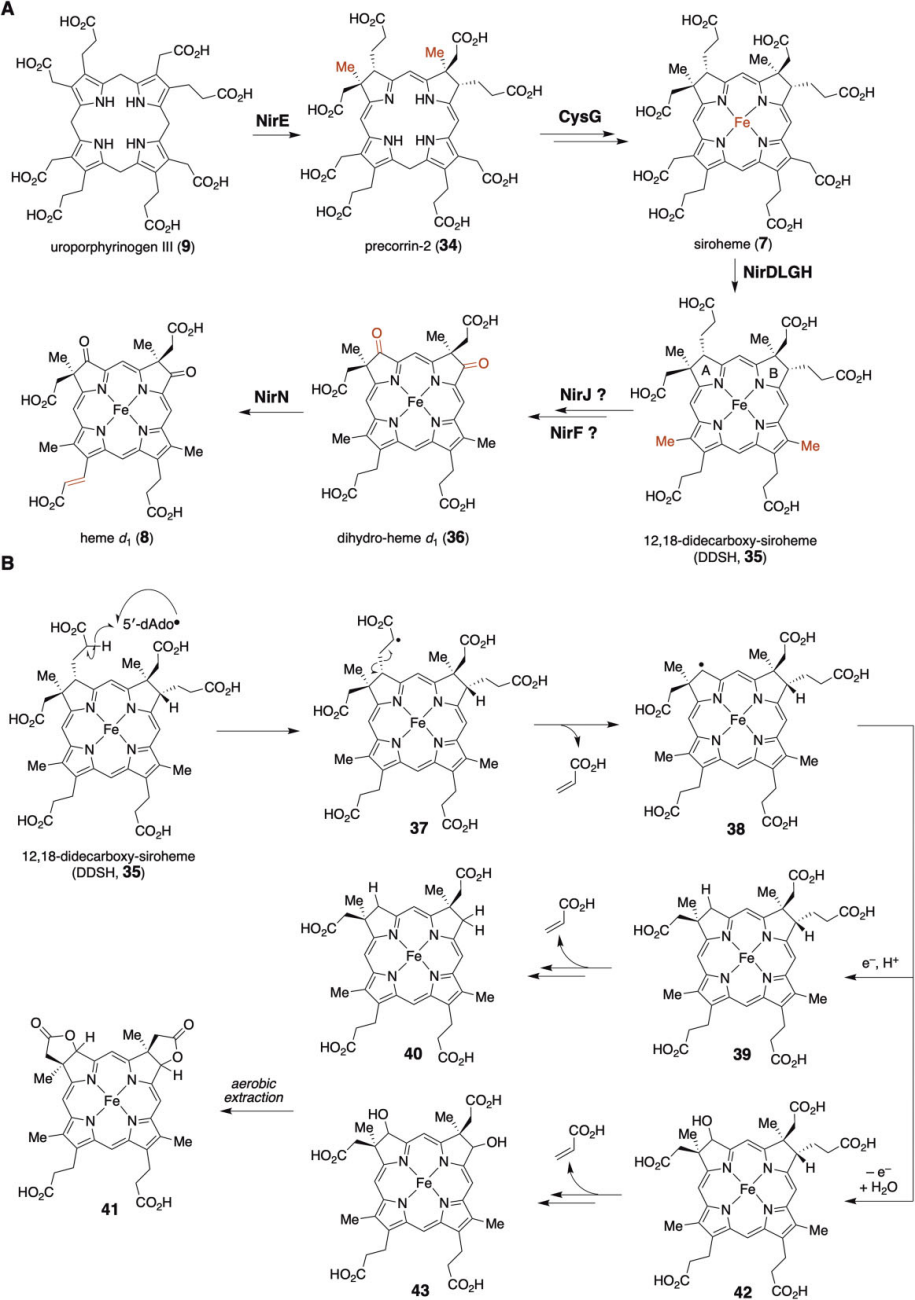
(A) Biosynthesis of heme *d*_1_ (**8**). (B) Proposed mechanism of NirJ.

Heme *d*_1_ (**8**) is a noncovalently bound cofactor of the cytochrome *cd*_1_ nitrite reductase NirS, which catalyzes the reduction of nitrite into nitrogen monoxide during the denitrification process in many denitrifying bacteria (Castiglione et al., 2012; Chang et al., 1986; Chang & Wu, 1986; Cutruzzolà et al., 2001, 2003; Fülöp et al., 1995; Li et al., 2013; Yamanaka et al., 1961). The structure of heme *d*_1_ (**8**) features an iron-containing dioxoisobacteriochlorin. The enzymes required for heme *d*_1_ (**8**) synthesis are encoded by the *nir* operon (de Boer et al., 1994; Kawasaki et al., 1995, 1997). The biosynthesis of heme *d*_1_ (**8**) begins with the NirE-catalyzed methylation of uroporphyrinogen III (**9**) (Storbeck et al., 2009, 2011; Zajicek et al., 2009), followed by macrocycle oxidation and ferrochelation by the multifunctional CysG (Warren et al., 1994) and decarboxylations of the acetate side chains on the C and D rings, catalyzed by NirDLGH (Bali et al., 2011; Haufschildt et al., 2014). However, the details of the biochemical conversion of 12,18-didecarboxy-siroheme (DDSH, **35**) to dihydro-heme *d*_1_ (**8**), the immediate precursor to heme *d*_1_ (**8**), remain unclear (Fig. 4A). NirJ is an annotated radical SAM enzyme that has been implicated as a key protein responsible for the cleavages of the propionate chains on the A and B rings in **35**, prior to the keto group introduction (Brindley et al., 2010). As a member of the radical SAM superfamily, NirJ contains the N-terminal cysteine-rich motif CX_3_CX_2_C, which binds to an [Fe_4_S_4_] cluster that is likely important for the reductive cleavage of SAM to initiate the radical chemistry (Brindley et al., 2010). In addition, the enzyme has another cysteine-rich motif, CX_2_CX_5_CX_20__–__21_C, which may coordinate an auxiliary [Fe_4_S_4_] cluster. An EPR analysis of anaerobically purified NirJ suggested that the [Fe_4_S_4_]^1+^ reductively cleaves SAM to generate 5′-dAdo• without a substrate (Brindley et al., 2010). The binding of two [Fe_4_S_4_] clusters to NirJ was also confirmed by iron and sulfur quantification, mutagenesis, and UV. When coexpressed with CysG and NirDLGH, NirJ co-purified with DDSH (**35**) with a small amount of dilactone **41**, suggesting that DDSH (**35**) is a substrate of NirJ (Boss et al., 2017). Indeed, when the NirJ/DDSH complex was reacted with dithionite and SAM, the consumption of DDSH was observed with the concomitant production of the dilactone **41** under the tested *in vitro* conditions. While the exact structure of the NirJ product is unclear, it is likely involved in the removal of the two propionate chains in DDSH (**35**) during heme *d*_1_ biosynthesis (Fig. 4A). NirF was also proposed to be involved in the oxo group introduction into dihydro-heme d1 (**36**), which is converted by NirN to form heme *d*_1_ (**8**) (Adamczack et al., 2014; Bali et al., 2010; Nicke et al., 2013). Although the crystal structure of NirF with dihydro-heme *d*_1_ (**36**) has been reported, its role in the heme *d*_1_ biosynthesis remains unclear (Klünemann et al., 2021).

Possible mechanisms for the NirJ reaction have been proposed, as shown in Fig. 4B (Boss et al., 2017). 5′-dAdo• may first abstract a H atom from the α position of one of the two propionate side chains in DDSH (**35**) to generate the corresponding substrate radical **37**, which then undergoes radical-mediated C–C bond cleavage to produce acrylate and the radical intermediate **38**. The fate of this radical is currently unknown. The intermediate **38** may be oxidized and hydrolyzed to yield an alcohol product, **42**. Alternatively, the radical may be reduced and protonated to generate **39**. A second round of NirJ catalysis, using another molecule of SAM, may be needed to cleave the other propionate side chain on the B ring. During the aerobic extraction for analysis, the lactonization of **43** or the oxidative cyclization of **40** may occur to produce the observed product, **41**. The amino acid sequence of NirJ shares similarity with that of AhbC (Layer et al., 2022); however, further examination will be necessary to determine the exact catalytic function of NirJ and its reaction mechanism.

## Biosynthesis of Tolyporphins, Bonellin, and Corallistins

**Fig. 5.**
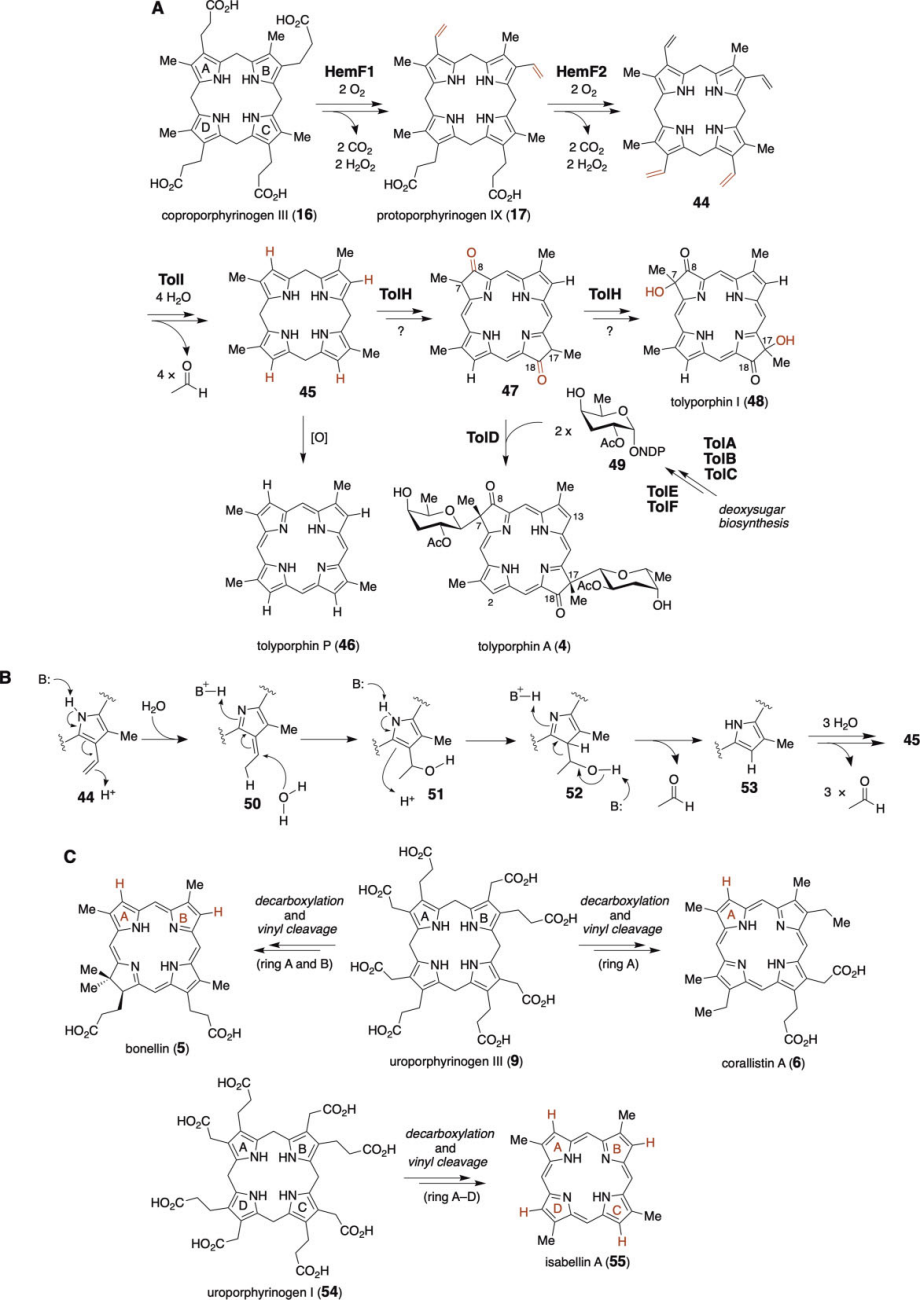
(A) Proposed biosynthetic pathway of tolyporphins. (B) Possible mechanism for the TolI-catalyzed vinyl group cleavage. (C) Biosynthesis of bonellin, corallistins, and isabellins.

Tolyporphins (such as **4, 46**, and **48**) are structurally unusual tetrapyrrole secondary metabolites produced by *Nostocales* cyanobacterium HT-58-2 cultures (Fig. 5A) (Gurr et al., 2019; Hughes et al., 2018; O'Donnell et al., 2021; Prinsep et al., 1992, 1995, 1998; Zhang et al., 2018). A total of 18 members have been isolated from the same cyanobacterial strain. The chemical structure of the most abundant member, tolyporphin A, features a dioxobacteriochlorin macrocycle decorated by two units of 2-*O*-acetylabequose via C-glycosidic bonds (Minehan et al., 1999; Prinsep et al., 1992). Notably, two of the pyrrole β sites (C2 and C13) of the macrocycle are unsubstituted, which is distinct from those in typical tetrapyrrole cofactors such as heme *b* (**1**) and chlorophyll *a* (**3**), while similar carbonyl functionalities at C8 and C18 can be found in heme *d*_1_ (**8**) as described above. Interestingly, the genome of the *Nostocales* cyanobacterium HT-58-2 strain contains two putative tolyporphin biosynthetic gene clusters, BGC-1 and BGC-2, in which many genes are duplicated (Hughes et al., 2017; Jin et al., 2021). The gene clusters encode some genes homologous to the heme biosynthetic genes (*hem*) and other genes unique to these clusters (*tol*). Although tolyporphin A has the dioxobacteriochlorin core, no radical SAM enzyme appears to be encoded by the gene clusters. Therefore, the two oxo groups in tolyporphin A would be introduced via a different biosynthetic mechanism from that of the heme *d*_1_ biosynthesis, which involves radical chemistry performed by NirJ.

HemF1, encoded by the *tol* gene cluster, exhibits high similarity to the canonical HemF, which catalyzes decarboxylations of the two propionate side chains on rings A and B of coproporphyrinogen III (**16**) (Fig. 5A) (Ushimaru et al., 2023). The *tol* gene cluster also encodes another HemF-like protein, HemF2, which only shows moderate similarity to HemF. *In vivo* and *in vitro* assays demonstrated that HemF1 has the same function as HemF: to convert coproporphyrinogen III (**16**) to protoporphyrinogen IX (**17**) via double decarboxylations (Fig. 5A). Instead, HemF2 decarboxylated the two remaining propionate side chains on rings C and D to generate the tetravinyl intermediate **44**. Comparisons of the predicted structures of HemF1 and HemF2 indicated that the active site pocket for substrate binding in HemF is smaller and more hydrophilic than that of HemF1. Thus, HemF2 appears to be a noncanonical protoporphyrinogen IX oxidase that evolved from HemF to accept **17** instead of **16**. A recent report showed that TolI, which was previously annotated as a hypothetical protein, removes all four vinyl groups from **44** to form **45** with the unsubstituted pyrrole β sites. TolI was characterized as an iron–sulfur cluster binding protein, although its exact function in the TolI reaction is unclear (Ushimaru et al., 2023).

A possible mechanism for the TolI-catalyzed vinyl group cleavage is shown in Fig. 5B (Ushimaru et al., 2023). First, a vinyl-substituted pyrrole moiety in **44** may be tautomerized to the conjugated imine form **50**. The alcohol **51** may be generated via 1,4-addition of water to **50**. The pyrrole moiety may undergo tautomerization again to generate a β-hydroxyl imine, which could undergo retro-aldol type C–C bond cleavage to generate **53** and acetaldehyde. Consistent with this mechanism, the detection of acetaldehyde as a by-product and the oxygen independence of the TolI catalysis were noted (Ushimaru et al., 2023). However, this hypothesis awaits further investigations for verification.

*In vivo* assays using *tolH*, encoding a putative cytochrome p450 enzyme, suggested that the gene product TolH is involved in the oxidation of the C8 and/or C18 positions, while its exact function is currently unknown (Fig. 5A) (Ushimaru et al., 2023). Tolyporphin A (**4**) may be derived from dioxobacteriochlorin **47** via C-glycosylation with the deoxysugar donor **49** catalyzed by TolD. Alternatively, further hydroxylations at C7 and C17 may be required to produce tolyporphin I (**48**). The simplest member, tolyporphin I (**46**), may be generated via the non-enzymatic or enzymatic oxidation of **45**. Although further biosynthetic investigations are needed to fully understand the mechanism by which tolyporphins are produced in nature, the work has provided new insights into how multiple C–C bond cleavage reactions are employed to substitute the carbon substituents at the β sites of the tetrapyrrole macrocycles with H atoms.

Bonellin (**5**) was isolated as a green pigment from a female green spoonworm (*Bonellia viridis*) (Fig. 5C) (Agius, 1979; Ballantine et al., 1980; Pelter et al., 1976). Bonellin (**5**) is regarded as a masculinization hormone as well as an agent for chemical defense (Agius et al., 1979; Cariello et al., 1980, 1982; Gauthier & de Nicola Giudici, 1983; Matthews et al., 1980). The structure of bonellin (**5**) resembles those of tolyporphins because they share unsubstituted pyrrole β sites in the tetrapyrrole macrocycle. Corallistins (such as **6**) are another group of tetrapyrrole natural products isolated from the Coral Sea demosponge *Corallistes* sp. (Fig. 5C) (D'Ambrosio et al., 1989, 1993). Recently, isabelline A (**55**) with a type-I methyl substitution pattern (D_4h_ symmetry) and derivatives thereof were isolated from the Western Australian sponge *Isabela* sp. (Sala et al., 2023). Considering the structural similarity between these compounds and tolyporphins, one might speculate that the biosynthesis of bonellin (**5**), corallistins (such as **6**), and isabellins (such as **55**) involves the decarboxylation of the propionate side chains (catalyzed by a HemF-like enzyme) of **9** or **54**, followed by vinyl group removal (catalyzed by a TolI-like enzyme) to generate the unsubstituted pyrrole carbons, in a mechanism similar to that in tolyporphin biosynthesis. However, these biosynthetic pathways have remained unexplored since their discovery, and further studies will be necessary to test these hypotheses.

## Conclusion

Tetrapyrroles represent a large group of metabolites with great structural diversity and biological significance. Among the many remarkable reactions in tetrapyrrole pathways, propionate side chain cleavages are key biosynthetic processes to tailor the peripheral regions of the common macrocycle precursors. Although diverse chemistries appear to be employed to remove the propionate side chains from tetrapyrroles under both aerobic and anaerobic conditions, the catalytic mechanisms of the C–C bond cleaving enzymes discussed in this review are only partially understood, and further structural and mechanistic enzyme characterizations will be needed. Considering the presence of many unusual tetrapyrrole cofactors and secondary metabolites in various organisms, we also expect that new enzymatic chemistries will continue to be uncovered in the biosynthetic pathways of this specific class of natural products.
